# Supplementary material for: Gene function prediction from bulk coexpression is bounded by cell-type-level signal
Source: bioRxiv. 2026 Sep 4:2026.08.31.748380. Preprint. [Version 1] doi: 10.64898/2026.08.31.748380 (PMC13684437; doi:10.64898/2026.08.31.748380)
Supplement: Supplementary file 1 [file NIHPP2026.08.31.748380v1-supplement-2.pdf]

bioRxiv preprint doi: https://doi.org/10.64898/2026.08.31.748380; this version posted September 4, 2026. The copyright holder for this preprint (which was not certified by peer review) is the author/funder, who has granted bioRxiv a license to display the preprint in perpetuity. It is made available under a CC-BY 4.0 International license.

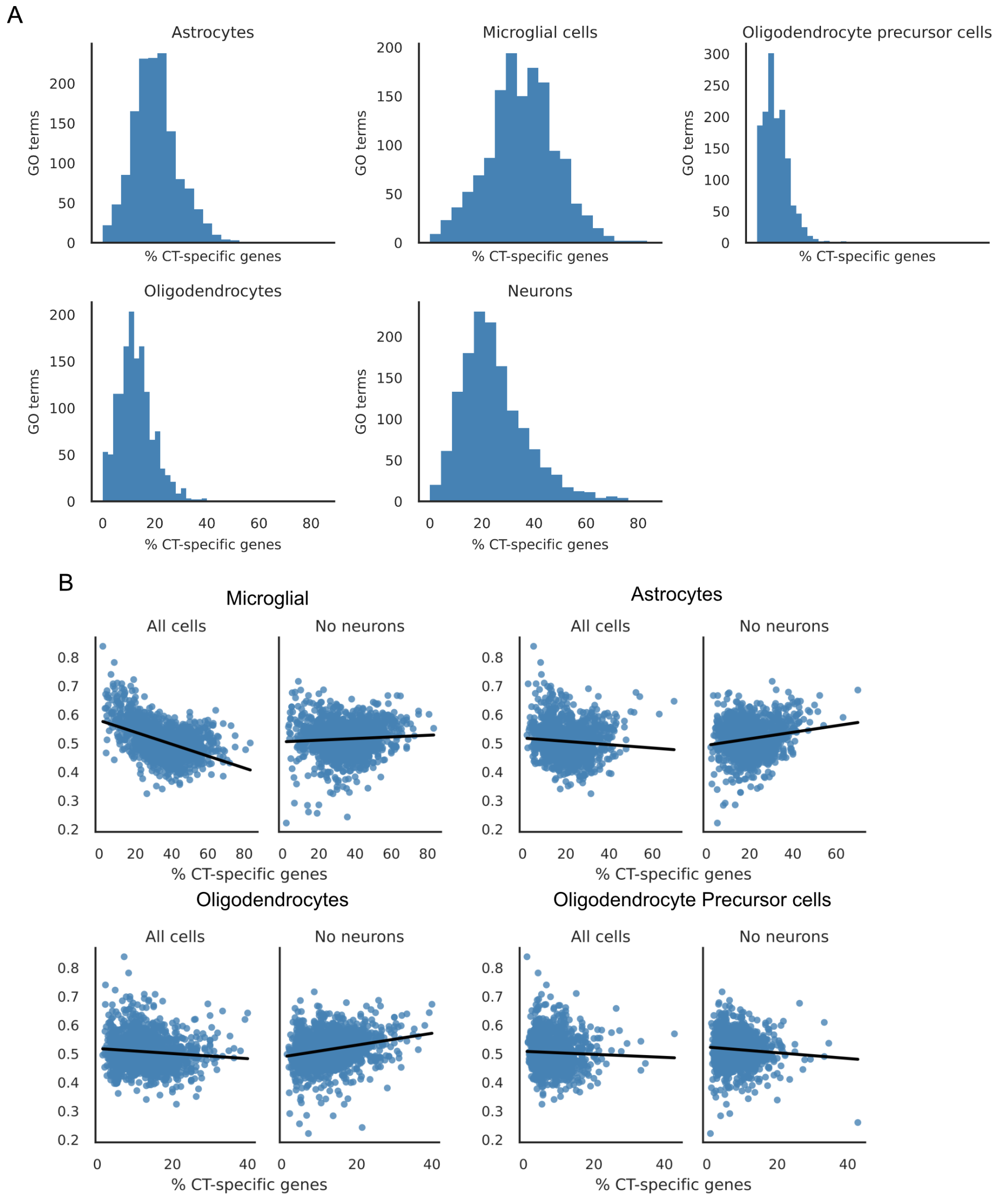

Supplementary Fig. S1 Cell-type-specific gene content across GO terms and its relationship to learnability for non-neuronal cell types. (**A**) Histograms of the percentage of cell-type-specific genes per GO term for each major brain cell type. (**B**) Relationship between GO term learnability and percentage of cell-type-specific genes for microglial cells, astrocytes, oligodendrocytes, and oligodendrocyte precursor cells, under two training conditions: all cell types included or excitatory neurons excluded.
